# Structure of the hypothetical DUF1811-family protein GK0453 from *Geobacillus kaustophilus* HTA426

**DOI:** 10.1107/S1744309113003369

**Published:** 2013-03-28

**Authors:** Balasundaram Padmanabhan, Yoshihiro Nakamura, Svetlana V. Antonyuk, Richard W. Strange, S. Samar Hasnain, Shigeyuki Yokoyama, Yoshitaka Bessho

**Affiliations:** a Department of Biophysics, National Institute of Mental Health and Neuro Sciences (NIMHANS), Bangalore 560 029, India; b RIKEN Systems and Structural Biology Center, 1-7-22 Suehiro-cho, Tsurumi-ku, Yokohama, Kanagawa 230-0045, Japan; c Molecular Biophysics Group, Institute of Integrative Biology, University of Liverpool, Liverpool L69 7ZB, England; d Laboratory of Structural Biology and Department of Biophysics and Biochemistry, Graduate School of Science, The University of Tokyo, 7-3-1 Hongo, Bunkyo-ku, Tokyo 113-0033, Japan; e RIKEN SPring-8 Center, Harima Institute, 1-1-1 Kouto, Sayo, Hyogo 679-5148, Japan

**Keywords:** DUF1811, *Geobacillus kaustophilus*, helix–turn–helix motif, β-barrel domain

## Abstract

The crystal structure of a conserved hypothetical protein, GK0453, from *Geobacillus kaustophilus* has been determined to 2.2 Å resolution. The crystal belonged to space group *P*4_3_2_1_2, with unit-cell parameters *a* = *b* = 75.69, *c* = 64.18 Å. The structure was determined by the molecular-replacement method and was refined to a final *R* factor of 22.6% (*R*_free_ = 26.3%). Based on structural homology, the GK0453 protein possesses two independent binding sites and hence it may simultaneously interact with two proteins or with a protein and a nucleic acid.

## Abstract 2

The gene encoding the hypothetical DUF1811-family protein GK0453 from *G. kaustophilus* was cloned and expressed. The crystal structure of the protein was determined by the molecular-replacement method and was refined to 2.2 Å resolution.

## 1. Introduction   

As part of the RIKEN Structural Genomics Initiative (RSGI) project, in collaboration with UK Structural Genomics, we selected the hypothetical protein GK0453 (13 kDa, 113 residues) from *Geo­bacillus kaustophilus* HTA426 to predict its function from analysis of its crystal structure. The GK0453 protein is a member of the DUF1811 family in the Pfam database (Bateman *et al.*, 2002 ▶). *G. kaustophilus*, from the Bacillaceae family, was isolated from deep-sea sediment from the Mariana Trench (Takami *et al.*, 1997 ▶). It is an aerobic, endospore-forming, Gram-positive bacterium that grows optimally at 333 K, with an upper temperature limit of 347 K (Takami *et al.*, 2004 ▶). There are 174 uncharacterized proteins in the DUF1811 family, and many are from *Bacillus* and *Staphylococcus* species that are known to cause a wide variety of diseases such as nosocomial infections. Thus, the proteins in this family may represent potential drug targets for highly selective bactericides or novel chemotherapies for these pathogens. The crystal structure of YfhH from *B. subtilis*, which belongs to this family, has been determined (PDB entry 1sf9; Midwest Center for Structural Genomics, unpublished work); however, the function of this protein is still unclear. Here, we describe the crystal structure of the hypothetical DUF1811-family protein GK0453 from *G. kaustophilus* and discuss its function based on structural homology.

## 2. Methods and materials   

### 2.1. Cloning, expression and purification   

The gene encoding the GK0453 protein (gi:56418988) was amplified *via* PCR using *G. kaustophilus* HTA426 genomic DNA and was cloned into the pET-15b expression vector (Merck Novagen, Darmstadt, Germany). The tobacco etch virus (TEV) protease recognition sequence was inserted in the N-terminal tag region of the expression vector, which was then introduced into the *Escherichia coli* Rosetta (DE3) strain (Merck Novagen, Darmstadt, Germany). The recombinant strain was cultured in 5 l LB medium containing 30 µg ml^−1^ chloramphenicol and 50 µg ml^−1^ ampicillin. The harvested cells (23.3 g) were lysed by sonication on ice in 35 ml of 20 m*M* Tris–HCl buffer pH 8.0 containing 500 m*M* NaCl, 5 m*M* β-mercaptoethanol and 1 m*M* phenylmethylsulfonyl fluoride. The cell lysate was heat-treated at 343 K for 13 min and then centrifuged at 15 000*g* for 30 min at 277 K. The supernatant was applied onto a HisTrap HP column (GE Healthcare Biosciences) equilibrated with 20 m*M* Tris–HCl buffer pH 8.0 containing 500 m*M* NaCl and 20 m*M* imidazole and eluted with a linear (20–500 m*M*) gradient of imidazole. The target sample, which eluted in the 500 m*M* imidazole fraction, was collected and applied onto a HiLoad 16/60 Superdex 200 pg column (GE Healthcare Biosciences) equilibrated with 20 m*M* Tris–HCl buffer pH 8.0 containing 200 m*M* NaCl and 20 m*M* imidazole. The eluted fractions containing the target sample were collected and treated with TEV protease at 303 K for 60 min. The sample was then applied onto a HisTrap HP column (GE Healthcare Biosciences) equilibrated with 20 m*M* Tris–HCl buffer pH 8.0 containing 500 m*M* NaCl and 20 m*M* imidazole. The flowthrough fraction was collected and desalted by fractionation on a HiPrep 26/10 column (GE Healthcare Biosciences) with 20 m*M* Tris–HCl buffer pH 8.0 containing 200 m*M* NaCl. The protein sample was analyzed by SDS–PAGE and its identity was confirmed by N-terminal amino-acid sequencing. After concentration to 28.5 mg ml^−1^ by ultrafiltration, the protein yield was 42.8 mg from 23.3 g of cells.

### 2.2. Protein crystallization, data collection and processing   

**Figure 1:**
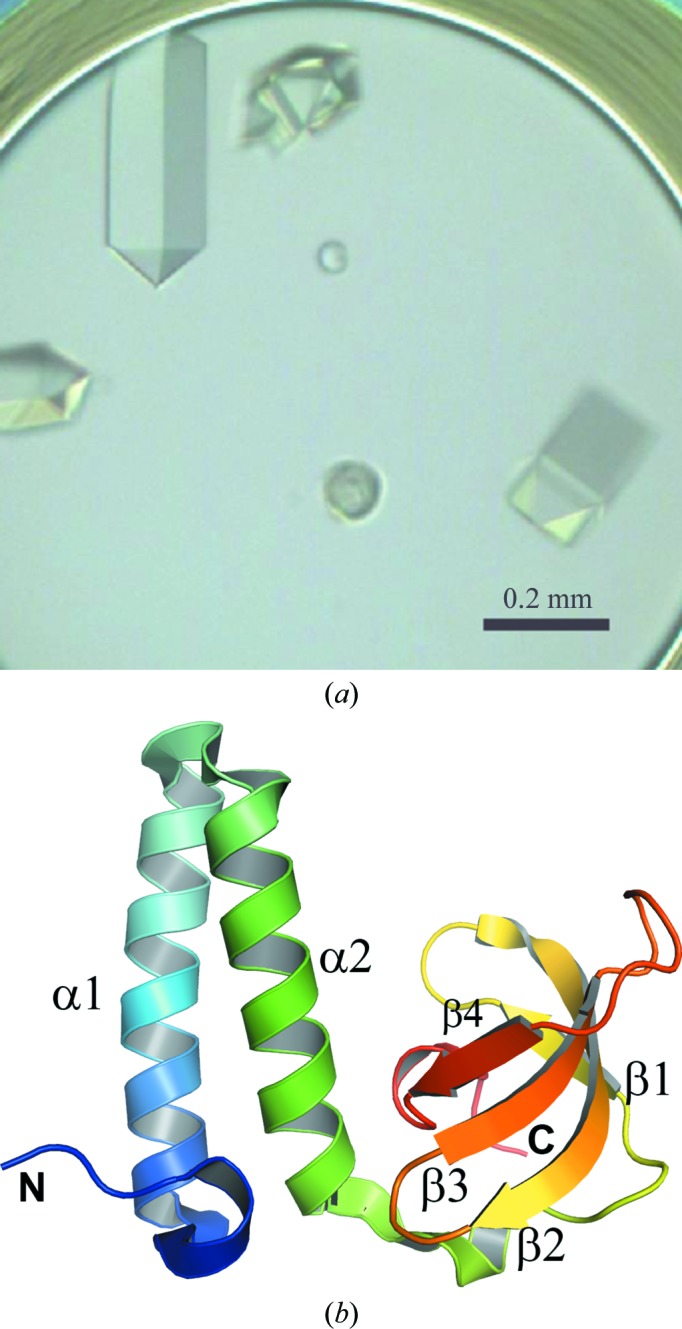
The structure of GK0453 from *G. kaustophilus*. (*a*) Crystals of the GK0453 protein. (*b*) Cartoon representation of the tertiary structure of GK0453 coloured in a rainbow ramp from blue at the N-terminus to red at the C-terminus. All figures were produced with *PyMOL* (Schrödinger) unless mentioned otherwise.

Crystallization was performed by the microbatch-under-oil method at 291 K. A 0.5 µl aliquot of crystallization reagent was mixed with 0.5 µl of the 28.5 mg ml^−1^ protein solution and was covered with 15 µl of silicone and paraffin oil. In the initial screening, small crystals appeared in a drop composed of 0.1 *M* Tris–HCl buffer pH 8.5 containing 20%(*w*/*v*) PEG MME 2000 and 0.01 *M* nickel(II) chloride hexahydrate (Crystal Screen 2 condition No. 45; Hampton Research). After optimization, large crystals were obtained from a crystallization reagent consisting of 0.1 *M* Tris–HCl buffer pH 8.1 containing 13.3%(*w*/*v*) PEG MME 2000 and 0.01 *M* NiCl_2_. Crystals suitable for X-ray data collection appeared within 1 d and reached dimensions of 0.42 × 0.15 × 0.12 mm (Fig. 1 ▶ *a*). The crystals were flash-cooled in a nitrogen-gas stream at 100 K using 10%(*v*/*v*) glycerol as a cryoprotectant. An X-ray diffraction data set was collected using a MAR Mosaic 225 CCD detector on beamline PX10.1 at the Daresbury Synchrotron Radiation Source (SRS), England. The data were integrated and scaled using the *HKL*-2000 software package. The data-reduction statistics are summarized in Table 1 ▶.

### 2.3. Structure determination and refinement   

The crystal structure of GK0453 was determined by the molecular-replacement method, using the YfhH protein structure as a search model (PDB entry 1sf9; Midwest Center for Structural Genomics, unpublished work). The program *MOLREP* from the *CCP*4 suite (Winn *et al.*, 2011 ▶) was used for structure determination. It generated a distinct peak with an *R* factor of 48.9% and a correlation coefficient of 45.1% for data in the resolution range 20–4 Å. The structure unambiguously revealed that the crystal belonged to space group *P*4_3_2_1_2 and contained one molecule in the asymmetric unit. The model was refined with *CNS* (Brünger *et al.*, 1998 ▶) and several rounds of manual fitting and re-fitting were performed using the program *O* (Jones *et al.*, 1991 ▶), with careful inspection of the 2*F*_o_ − *F*_c_, *F*_o_ − *F*_c_ and OMIT electron-density maps. The final *R* factor and *R*_free_ were 22.6 and 26.3%, respectively, at 2.2 Å resolution. In the final structure, four residues (residues 1–4) in the N-terminal region and five residues in the C-terminal region (residues 109–113) were absent owing to poor electron density in these regions. The stereochemistry of the GK0453 structure was good as assessed by *MolProbity* (Chen *et al.*, 2010 ▶). The structure was deposited in the PDB under accession code 2yxy. The refinement statistics are summarized in Table 1 ▶.

## 3. Results and discussion   

### 3.1. Overall structure   

The overall tertiary structure of *G. kaustophilus* GK0453 consists of two small domains at the N- and C-terminal regions (Fig. 1 ▶ *b*). The N-terminal region contains a helix–turn–helix motif (α1, Lys14–Met34; α2, Val37–Tyr53). The C-terminal domain possesses a β-­barrel-like structure with four β-strands (β1, Glu64–Ile68; β2, Ala71–Lys82; β3, Phe85–Arg90; β4, Glu98–Pro101). A long loop containing a 3_10_-helix (Pro57–Asp59) connects the N- and C-­terminal domains (Figs. 1 ▶ *b* and 2 ▶ *a*).

### 3.2. Structure comparison and functional prediction   

**Figure 2:**
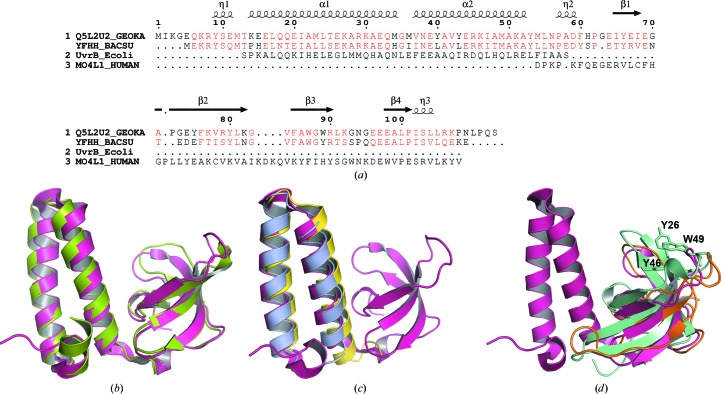
Structural comparisons of GK0453. (*a*) Sequence alignment of GK0453 (Q5L2U2_GEOKA) with the hypothetical protein YfhH (YFHH_BACSU) and representative structurally similar proteins UvrB (UvrB_Ecoli; amino acids 628–673) and the MRG15 chromodomain protein (MO4L1_HUMAN; amino acids 6–65) corresponding to the N- and C-terminal regions of GK0453, respectively. The secondary-structure elements of GK0453 are indicated above the alignment and residues that are similar between GK0453 and YfhH are coloured red. The figure was generated by *ESPript* (Gouet *et al.*, 1999 ▶). Superimpositions are shown of (*b*) GK0453 (pink) on the YfhH protein (green), (*c*) the N-terminal region of GK0453 on the C-terminal domain of UvrB (light blue) and on the Rab-binding domain of rabenosyn-5 (yellow) and (*d*) the C-terminal region of GK0453 on the MRG15 chromodomain (cyan) and on the type II dihydrofolate reductase DHFR (orange). The methylated histone-tail recognizing residues Tyr26, Tyr46 and Trp49 in the MRG15 chromodomain are depicted by sticks.

A *DALI* (Holm & Rosenström, 2010 ▶) search was performed for the GK0453 structure to identify structural homologues within the RCSB PDB. The search revealed that the GK0453 structure is very similar to that of the hypothetical protein YfhH (60.8% sequence identity; PDB entry 1sf9). Superimposition of the GK0453 structure on the YfhH structure yielded a *Z*-score of 15.4 and an r.m.s.d. of 1.8 Å for 101 C^α^ atoms (Figs. 2 ▶ *a* and 2 ▶ *b*). All other results from the search showed that the structural similarity occurred within the distinct domains of either the 44-amino-acid N-­terminal region or the 54-amino-acid C-terminal region. The N-­terminal region of GK0453 is structurally homologous to the C-­terminal domain of UvrB (PDB entry 1qoj; 19% identity; *Z*-score of 7.3 and r.m.s.d. of 0.6 Å for 43 C^α^ atoms; Sohi *et al.*, 2000 ▶) and to the minimal Rab-binding domain of rabenosyn-5 (PDB entry 1z0j; 23% identity; *Z*-score of 6.9 and r.m.s.d. of 1.7 Å for 48 C^α^ atoms; Eathiraj *et al.*, 2005 ▶) (Fig. 2 ▶ *c*). The UvrB C-terminal domain interacts with the UvrC C-terminal domain during excision repair in *E. coli*, and the UvrBC complex is part of the UvrABC endonuclease system, which catalyzes DNA damage repair (Sohi *et al.*, 2000 ▶). The other protein family, including rabenosyn-5, selectively recognizes distinct subunits of Rab GTPases exclusively through interactions with the switch and inter-switch regions in the helix–turn–helix motif (Eathiraj *et al.*, 2005 ▶). A similar structural feature is also observed in several PDB structures of proteins that interact with 23S ribosomal RNA. For example, the ribosomal protein L29 (PDB entry 2gya, chain *W*; Mitra *et al.*, 2006 ▶) yielded 20% identity and a *Z*-score of 7.2 and an r.m.s.d. of 1.7 Å for 50 C^α^ atoms. This ribosomal subunit protein, which contains a helix–turn–helix motif, extensively interacts with the 23S ribosomal RNA. Hence, we speculated from this analysis that the N-­terminal region of GK0453 may be involved in a protein–protein or a protein–nucleic acid interaction.

In contrast, the C-terminal domain of GK0453 is structurally homologous to those of the MRG15 chromodomain (PDB entry 2f5k; 22% sequence identity; *Z*-score of 6.7 and r.m.s.d. of 2.3 Å for 54 C^α^ atoms; Zhang *et al.*, 2006 ▶), the nuclear protein KIN17 (PDB entry 2ckk, chain *A*; 16% sequence identity; *Z*-score of 6.4 and r.m.s.d. of 2.2 Å for 52 C^α^ atoms; le Maire *et al.*, 2006 ▶) and type II R-plasmid-encoded R67 dihydrofolate reductase (R67 DHFR; PDB entry 2rh2, chain *A*; 12% identity; *Z*-­score of 6.5 and r.m.s.d. of 2.7 Å for 51 C^α^ atoms; Krahn *et al.*, 2007) (Fig. 2 ▶ *d*).

**Figure 3:**
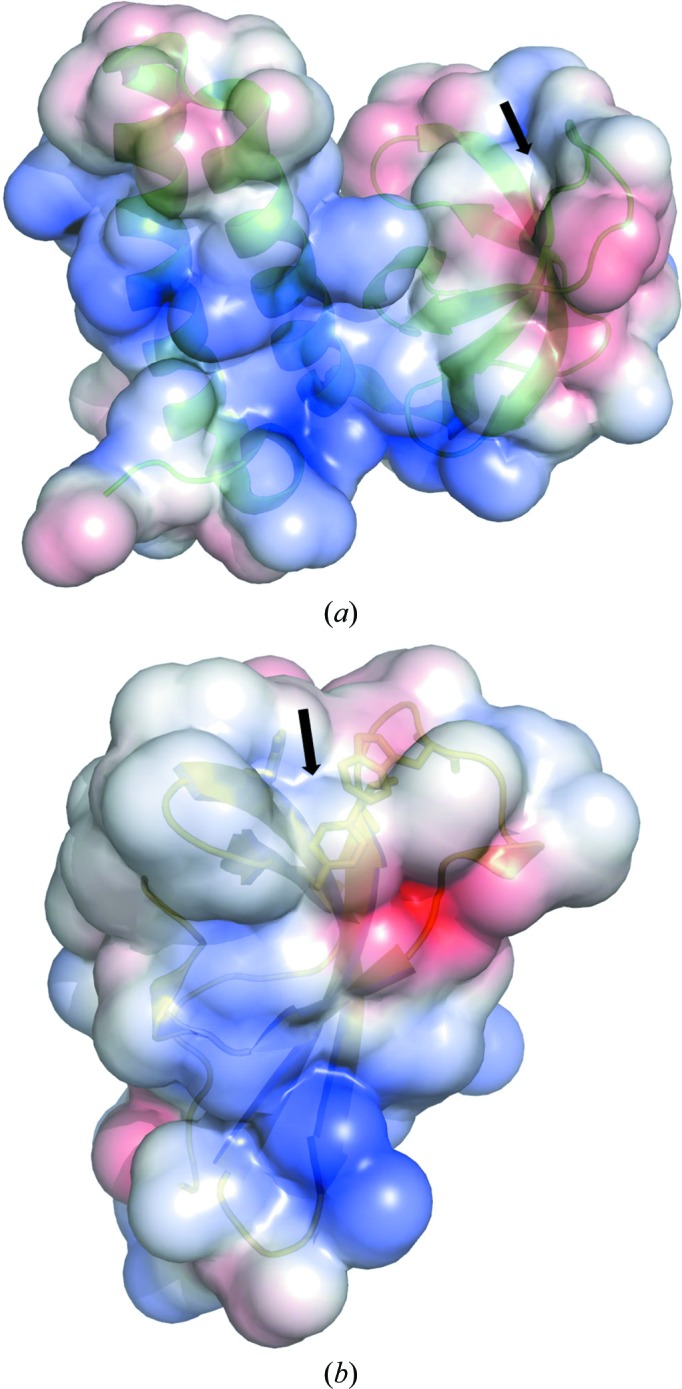
The electrostatic surface potentials of (*a*) the GK0453 protein and (*b*) the MRG15 chromodomain protein. The arrows indicate the similar hydrophobic environments present in both GK0453 and MRG15. The surface is coloured red and blue for potential values below −5*k*_B_ *T* and above +5*k*_B_ *T*, respectively, where *k*_B_ is the Boltzmann constant and *T* is room temperature.

The R67 DHFR protein is an NADPH-dependent enzyme that catalyzes the reduction of dihydrofolate (DHF) to tetrahydrofolate (THF). THF is essential for the synthesis of thymidylate, purine nucleosides, methionine and other metabolic intermediates (Krahn *et al.*, 2007 ▶). Since the functional tetramerization and the critical residues for enzymatic function are absent in GK0453, it is unlikely that GK0453 possesses an activity similar to that of DHFR. The human KIN17 protein is an essential nuclear component that plays a critical role in maintaining the integrity of the human global genome-repair machinery. The SH3-like β-barrel domain of KIN17 has been shown to interact with RNA (le Maire *et al.*, 2006 ▶). As GK0453 possesses a hydrophobic environment in the corresponding region, it is unlikely that GK0453 interacts with RNA or DNA through this region (Fig. 3 ▶ *a*).

The MRG15 chromodomain participates in chromatin remodelling and transcription regulation by interacting with the methylated histone tail of the nucleosome (Zhang *et al.*, 2006 ▶; Steiner *et al.*, 2002 ▶). The β-barrel core forms a hydrophobic pocket containing three conserved residues, Tyr26, Tyr46 and Trp49, as a potential binding site for interaction with the methylated histone tail (Fig. 2 ▶ *d*). The domain bearing these residues, which are responsible for recognizing the methylated histone tail, is absent in GK0453; however, the GK0453 and MRG15 chromodomain structures both possess similar hydrophobic environments (Fig. 3 ▶). However, the structural analysis suggested that the β-barrel domain of GK0453 may also be involved in protein–protein interactions with an unknown function.

In conclusion, the crystal structure of the hypothetical protein GK0453 revealed two small domains: a helix–turn–helix motif at the N-terminal region and an SH3-like β-barrel structure at the C-­terminal region. Based on structural comparisons, we speculate that the GK0453 protein may simultaneously interact with two proteins or with a protein and a nucleic acid to exert its unknown function.

## Supplementary Material

PDB reference: GK0453, 2yxy

## Figures and Tables

**Table 1:** Summary of data-collection and refinement statistics Values in parentheses are for the highest resolution shell.

Data collection	
Source	SRS PX10.1
Wavelength ()	1.117
Space group	*P*4_3_2_1_2
Unit-cell parameters ()	*a* = *b* = 75.7, *c* = 64.2
Resolution ()	20.02.2
Completeness (%)	99.8 (99.6)
Multiplicity	10.3 (10.6)
*R*_merge_ † (%)	7.8 (27.9)
Refinement statistics	
No. of molecules in asymmetric unit	1
Resolution limits ()	20.02.2
cutoff	0
No. of reflections	9710
*R* factor‡/*R*_free_ § (%)	22.6/26.3
No. of protein residues	104
No. of water molecules	170
R.m.s. deviations	
Bond lengths ()	0.011
Bond angles ()	1.4

† *R*_merge_ = 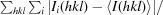 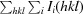.

‡ *R* = 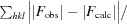 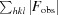, where *F*_obs_ and *F*_calc_ are the observed and calculated structure factors, respectively.

§ *R*_free_ was calculated with 5% of data that were omitted from refinement.

## References

[bb1] Bateman, A., Birney, E., Cerruti, L., Durbin, R., Etwiller, L., Eddy, S. R., Griffiths-Jones, S., Howe, K. L., Marshall, M. & Sonnhammer, E. L. (2002). *Nucleic Acids Res.* **30**, 276–280. 10.1093/nar/30.1.276 PMC99071 11752314

[bb2] Brünger, A. T., Adams, P. D., Clore, G. M., DeLano, W. L., Gros, P., Grosse-Kunstleve, R. W., Jiang, J.-S., Kuszewski, J., Nilges, M., Pannu, N. S., Read, R. J., Rice, L. M., Simonson, T. & Warren, G. L. (1998). *Acta Cryst.* D**54**, 905–921. 10.1107/s0907444998003254 9757107

[bb3] Chen, V. B., Arendall, W. B., Headd, J. J., Keedy, D. A., Immormino, R. M., Kapral, G. J., Murray, L. W., Richardson, J. S. & Richardson, D. C. (2010). *Acta Cryst.* D**66**, 12–21. 10.1107/S0907444909042073 PMC2803126 20057044

[bb5] Eathiraj, S., Pan, X., Ritacco, C. & Lambright, D. G. (2005). *Nature (London)*, **436**, 415–419. 10.1038/nature03798 PMC1360218 16034420

[bb6] Gouet, P., Courcelle, E., Stuart, D. I. & Métoz, F. (1999). *Bioinformatics*, **15**, 305–308. 10.1093/bioinformatics/15.4.305 10320398

[bb7] Holm, L. & Rosenström, P. (2010). *Nucleic Acids Res.* **38**, W545–W549. 10.1093/nar/gkq366 PMC2896194 20457744

[bb8] Jones, T. A., Zou, J.-Y., Cowan, S. W. & Kjeldgaard, M. (1991). *Acta Cryst.* A**47**, 110–119. 10.1107/s0108767390010224 2025413

[bb9] Krahn, J. M., Jackson, M. R., DeRose, E. F., Howell, E. E. & London, R. E. (2007). *Biochemistry*, **46**, 14878–14888. 10.1021/bi701532r PMC3743094 18052202

[bb10] Maire, A. le, Schiltz, M., Stura, E. A., Pinon-Lataillade, G., Couprie, J., Moutiez, M., Gondry, M., Angulo, J. F. & Zinn-Justin, S. (2006). *J. Mol. Biol.* **364**, 764–776. 10.1016/j.jmb.2006.09.033 17045609

[bb16] Mitra, K., Schaffitzel, C., Fabiola, F., Chapman, M. S., Ban, N. & Frank, J. (2006). *Mol. Cell*, **22**, 533–543. 10.1016/j.molcel.2006.05.003 16713583

[bb11] Sohi, M., Alexandrovich, A., Moolenaar, G., Visse, R., Goosen, N., Vernede, X., Fontecilla-Camps, J. C., Champness, J. & Sanderson, M. R. (2000). *FEBS Lett.* **465**, 161–164. 10.1016/s0014-5793(99)01690-7 10631326

[bb12] Steiner, T., Kaiser, J. T., Marinkoviç, S., Huber, R. & Wahl, M. C. (2002). *EMBO J.* **21**, 4641–4653. 10.1093/emboj/cdf455 PMC126194 12198166

[bb13] Takami, H., Inoue, A., Fuji, F. & Horikoshi, K. (1997). *FEMS Microbiol. Lett.* **152**, 279–285. 10.1111/j.1574-6968.1997.tb10440.x 9231422

[bb14] Takami, H., Nishi, S., Lu, J., Shimamura, S. & Takaki, Y. (2004). *Extremophiles*, **8**, 351–356. 10.1007/s00792-004-0394-3 15168170

[bb4] Winn, M. D. *et al.* (2011). *Acta Cryst.* D**67**, 235–242.

[bb15] Zhang, P., Du, J., Sun, B., Dong, X., Xu, G., Zhou, J., Huang, Q., Liu, Q., Hao, Q. & Ding, J. (2006). *Nucleic Acids Res.* **34**, 6621–6628. 10.1093/nar/gkl989 PMC1747190 17135209

